# Trauma-informed Care Interventions in Emergency Medicine: A Systematic Review

**DOI:** 10.5811/westjem.2022.1.53674

**Published:** 2022-04-13

**Authors:** Taylor Brown, Henry Ashworth, Michelle Bass, Eve Rittenberg, Nomi Levy-Carrick, Samara Grossman, Annie Lewis-O’Connor, Hanni Stoklosa

**Affiliations:** * Beth Israel Deaconess Medical Center, Department of Emergency Medicine, Boston, Massachusetts; † Harvard Medical School, Boston, Massachusetts; ‡ Harvard Medical School, Countway Library, Boston, Massachusetts; § Brigham and Women’s Hospital, Department of Medicine, Boston, Massachusetts; ¶ Brigham and Women’s Hospital, Department of Psychiatry, Boston, Massachusetts; || Brigham and Women’s Hospital, Department of Emergency Medicine, Boston, Massachusetts

## Abstract

**Introduction:**

Trauma exposure is a highly prevalent experience for patients and clinicians in emergency medicine (EM). Trauma-informed care (TIC) is an effective framework to mitigate the negative health impacts of trauma. This systematic review synthesizes the range of TIC interventions in EM, with a focus on patient and clinician outcomes, and identifies gaps in the current research on implementing TIC.

**Methods:**

The study was registered with PROSPERO (CRD42020205182). We systematically searched peer-reviewed journals and abstracts in the PubMed, EMBASE (Elsevier), PsycINFO (EBSCO), Social Services Abstract (ProQuest), and CINAHL (EBSCO) databases from 1990 onward on August 12, 2020. We analyzed studies describing explicit TIC interventions in the ED setting using inductive qualitative content analysis to identify recurrent themes and identify unique trauma-informed interventions in each study. Studies not explicitly citing TIC were excluded. Studies were assessed for bias using the Newcastle-Ottawa criteria and Critical Appraisal Skills Programme (CASP) Checklist.

**Results:**

We identified a total of 1,372 studies and abstracts, with 10 meeting inclusion criteria for final analysis. Themes within TIC interventions that emerged included educational interventions, collaborations with allied health professionals and community organizations, and patient and clinician safety interventions. Educational interventions included lectures, online modules, and standardized patient exercises. Collaborations with community organizations focused on addressing social determinants of health. All interventions suggested a positive impact from TIC on either clinicians or patients, but outcomes data remain limited.

**Conclusion:**

Trauma-informed care is a nascent field in EM with limited operationalization of TIC approaches. Future studies with patient and clinician outcomes analyzing universal TIC precautions and systems-level interventions are needed.

## INTRODUCTION

### Background

Trauma exposure is a highly prevalent experience in the emergency department (ED) for both patients and clinicians.^1^–^6^ The Substance Abuse and Mental Health Services Administration (SAMHSA) defines trauma as “an event, series of events, or set of circumstances that is experienced by an individual as physically or emotionally harmful or life-threatening and that has lasting adverse effects on the individual’s functioning and mental, physical, social, emotional, or spiritual well-being.”^7^ This definition of trauma encompasses experiences that range from individual (eg, car accident, death of a loved one), to interpersonal (eg, interpersonal violence [IPV], discrimination, abuse), to societal (eg, natural disasters, pandemics, terrorist attacks). Newer publications have expanded this definition to explicitly address structural trauma (eg, racism, sexism).^8^

Patients frequently present to the ED with the types of trauma defined above: individual (medical traumas/injuries); IPV; and societal traumas (gun violence and community violence). In the United States, the yearly incidence of these events range from 1.7 million ED visits for assault-related injury^1^ to 88,000 due to firearm-related injuries,^2^ and over 28,000 ED visits attributed to IPV.^3^ Patients presenting with acute trauma often are survivors of previous traumatic experiences; a survey of survivors of community violence participating in a hospital-based violence intervention program found that 100% of participants reported at least one adverse childhood experience.^9^ These previous traumatic experiences are not equally distributed, with those self-identified as female, American Indian/Alaskan Native, and Black being more likely to experience several types of adverse childhood experience than those self-identified as male or White.^10^

For some survivors of trauma, the experience of the ED may be re-traumatizing or trigger past experiences.^11^ Survivors of trauma may experience emotional dysregulation (ie, trouble controlling strong emotions) or hypervigilance (ie, increased threat perception and reactivity).^12^ The close interplay between executive functioning and emotional regulation may impact both the patient and the care team’s navigation of the encounter.^12^ Similarly, hypervigilance could make the often-hectic environment of the ED, as well as interventional procedures, harder to tolerate.^12^

The ED setting, by virtue of its emergency-level care, presents multiple potential sources for both direct and secondary trauma (ie, indirect exposure to traumatic events) to clinicians and non-clinical staff. The COVID-19 pandemic demonstrated the toll secondary trauma exposure can have on frontline healthcare workers and staff.^4^ Staff practicing in the ED also experience high rates of workplace violence (ie, direct trauma). 5,6 The combination of direct trauma and secondary trauma likely contributes to the high rates of post-traumatic stress disorder (PTSD) and secondary traumatic stress (STS) experienced by emergency clinicians. About 11.9–16.8% of emergency physicians screen positive for PTSD and STS symptoms at any one time,^13^–^18^ and these rates may be even higher in emergency nurses with 33–64% of nursing staff screening positive for at least one symptom of STS.^19^–^21^ There is evidence to suggest that non-clinical staff also experience STS from witnessing acute care.^22^

### Importance

Trauma-informed care (TIC) is a framework that aims to prevent re-traumatization in the healthcare setting and promote resilience for both patient and clinicians.^23^ It is based on six principles: 1) safety; 2) trustworthiness and transparency; 3) peer support; 4) collaboration and mutuality; 5) empowerment, voice, and choice; and 6) cultural, historical, and gender issues.^7^ Trauma-informed care is increasingly being adopted as an approach to clinical care in both primary and specialty care, including emergency medicine (EM).^23^–^28^ In 2012, the US Attorney General National Task Force on Children Exposed to Violence called for all EDs to provide TIC, and for all clinicians interacting with patients experiencing trauma to be trained in TIC.^29^ Trauma-informed care has been shown to be a cost-effective intervention with clinical benefits to patients and job satisfaction benefits to staff.^30^–^33^ However, despite the immense burden of trauma seen in the ED and the benefits of TIC for patients and clinicians, TIC remains a nascent field within EM.

### Goals of This Investigation

This review will synthesize evidence on TIC interventions in EM to describe the following research aims: the breadth of TIC interventions being pursued in the physical ED setting; the potential benefits to patients of TIC interventions in the ED; the potential benefits to clinicians and non-clinical staff of TIC interventions in the ED; and to identify gaps in the current research on implementing TIC interventions in the ED.

## METHODS

### Search Strategy

We searched peer-reviewed journals and abstracts by searching the databases PubMed, EMBASE (Elsevier), PsycINFO (EBSCO), Social Services Abstract (ProQuest), and CINAHL (EBSCO). The searches included keywords and controlled vocabulary terms for the following concepts: the physical space of the ED; clinicians and staff in the ED; and TIC. A full description of search terms can be found in Appendix 1. The final protocol was registered with PROSPERO (CRD42020205182).

### Study Selection

Since TIC as a framework was developed in the 1990s, we included studies from 1990 onward to August 12, 2020, when databases were queried. We included any study that involved emergency clinicians (eg, physicians, nurses, nurse practitioners, and physician assistants) and non-clinical staff (eg, administrative staff, security staff, and environmental services staff). We included studies that examined the physical setting of the ED. The review included studies that reported on TIC interventions. Our study focused on TIC as a framework; therefore, studies had to mention TIC explicitly to be included. Studies that mentioned one element of TIC without referencing the framework were not included. A more detailed explanation of the TIC framework is included in Appendix 2. We defined the criteria for intervention broadly to include any explicit application of TIC. This included TIC related to the physical environment of the ED, TIC clinical care in the ED, TIC guiding policies of the ED, and any educational intervention that explicitly instructs on TIC.

**Figure 1:**
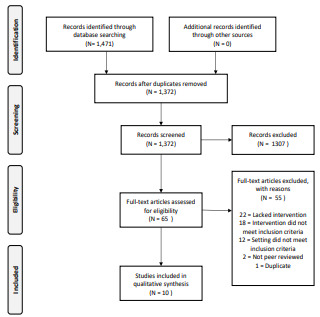
PRISMA flow diagram. *PRISMA*, Preferred Reporting Items for Systematic Reviews and Meta-Analyses; *TIC*, trauma-informed care.

Since TIC is a relatively new conceptual framework, we anticipated there would be few if any randomized controlled trials. We anticipated a breadth of outcomes with a broad definition of “intervention.” For this reason, we did not limit the study design. We excluded the following: non-peer reviewed literature; studies not published in English; studies that did not explicitly name TIC as a framework; studies that did not comment on the operationalization of specific interventions; and studies describing trauma-focused treatment for psychiatric symptoms of stress disorders. Studies not meeting our criteria were excluded in the title and abstract screening phase (Figure 1).

The medical librarian (MB) downloaded resulting citations to Covidence Systematic Review Software (Veritas Health Innovation, Melbourne, Australia) and removed duplicate citations. Next, two independent reviews (TB and HA) screened the titles and abstracts of the selected citations for inclusion or exclusion based on our pre-established criteria. When there was disagreement during this phase of screening, the result was included in the full-text review. Reviewers (TB and HA) then assessed full-text articles for inclusion. Conflicts were resolved by a third, more senior reviewer (HS). Risk-of-bias assessment was conducted (HA) using the Newcastle-Ottawa criteria for randomized control trials, cohort studies, and case studies. The Critical Appraisal Skills Programme (CASP) Checklist was used to assess qualitative research studies.

### Data Extraction & Analysis

We extracted data manually into Microsoft Excel (Microsoft Corporation, Redmond, WA). For each study we recorded 1) author and date, 2) country, 3) specific intervention, 4) study design, 5) study participants, 6) number of participants, 7) form of trauma, 8) facility type, 9) primary conclusion, and 10) secondary conclusion. Due to the anticipated heterogeneity of results and the early stage of implementation, we did not plan for meta-analysis. We instead chose qualitative thematic analysis across the studies with a focus on unique interventions such as described by Bendall and colleagues.^34^ Two independent reviewers (TB and HA) used NVivo12 software (QSR International, Melbourne, Australia) to code included studies. We used an inductive content analysis to identify recurrent themes and identify unique trauma-informed interventions in each study.

## RESULTS

A total of 1372 studies and abstracts were identified from our search. We excluded 1307 during the title and abstract screen. The majority of the excluded studies in this phase were not relevant as defined by the inclusion and exclusion criteria defined above (ie, trauma studies isolated to physical injury or trauma-focused psychiatric treatment). We assessed 65 articles during the full-text phase and excluded 55 studies, leaving 10 studies for inclusion. Reasons for exclusion during the full-text analysis are included in Figure 1.

Of the 10 studies included, five represent primarily educational interventions and five describe protocols or programs that operationalized a TIC framework. Full details of each article can be found in Table 1. Major quantitative and qualitative results are summarized in Table 2.

No studies were excluded during the risk-of bias assessment. Full results from the risk-of-bias assessment are included in Appendix 3. Themes emerging from the qualitative analysis of unique interventions included the following: education, collaboration, and safety. Our inter-rater reliability score was 0.89. A complete summary of interventions, including those not described fully in the analysis, appears in Table 3.

### Education

Seven papers included an educational component.^35^–^41^ Of the interventions collecting data, all reported effectiveness in increasing clinicians’ comfort and knowledge of TIC.^35^–^37^,^39^,^41^ Educational interventions ranged in length from 15 minutes^39^ to around eight hours^41^ and used a variety of mediums including in-person didactics,^35^–^38^,^40^,^41^ online modules,^39^ and standardized patient encounters.^36^ Prior to conducting an educational intervention, many sites conducted needs assessments.^35^–^37^,^39^ Chandramani et al found that clinicians lacked training and confidence in providing TIC to survivors of sexual assault and that they did not understand hospital policy or state laws relating to sexual assault.^36^ The authors incorporated these findings into subsequent educational interventions.

Educational content across the studies included trauma epidemiology and health impacts,^35^–^39^,^42^ trauma responses,^38^,^42^ and TIC clinical skills.^35^–^37^,^41^ All educational interventions focused on specific patient populations including survivors of sexual assault,^35^,^36^ community violence,^38^ human trafficking,^40^ pediatrics,^39^ and patients experiencing mental health crises.^37^,^41^ Two educational interventions collected patient outcomes data.^35^,^37^ One study showed a reduction in the number of patients subjected to restraint and reduced overall patient time in restraints among mental health patients following TIC education of clinicians in the ED.^37^ Another study showed an improvement in quality of service ratings and consistency of referrals among survivors of sexual assault.^35^

### Collaboration

Eight of the studies included in this review contained a collaboration as an intervention.^35^–^38^,^40^,^42^–^44^ Almost all reported how collaboration was important for the success of each intervention. Themes emerging within collaboration included collaboration across physician specialties,^36^,^40^,^44^ collaboration across allied health professions,^35^–^38^,^40^,^42^ collaboration with community organizations,^35^,^36^,^38^,^40^,^44^ and collaboration in arranging post-ED follow-up.^38^,^43^,^44^ Each of the collaborations identified a specific patient population including survivors of community violence,^38^,^44^ human trafficking,^40^ terrorist attacks,^42^ and pediatric mental health.^43^

The Healing Hurt People program described by Corbin et al connects survivors of community violence with a host of resources including “obtaining identification and health insurance, substance abuse treatment, post-traumatic stress treatment, healthcare, education, housing, job training and placement, legal assistance, transportation, counseling, and physical rehabilitation” through collaboration with social workers and community organizations.^38^ Collaborations with community organizations were vital to addressing social determinants of health including housing instability, food insecurity, and economic insecurity.^35^,^36^,^38^,^40^,^44^ In developing a protocol for survivors of human trafficking in the ED, Tiller et al collaborated with community organizations to provide survivors with a “list of resources for the patient beyond medical care such as emergency housing, legal assistance, and food pantries.”^40^ Collaborations with allied health professions were most often with social work^38^,^40^ and nursing.^36^,^37^,^42^ Several interventions collaborated with local law enforcement for bi-directional education.^35^,^40^

### Safety

Six papers detailed interventions operationalizing patient or staff safety using TIC.^37^,^38^,^40^–^43^ Themes emerging within safety included the following: safety precautions for patient’s emotional and physical wellbeing; interventions to ensure staff’s safety; and safety assessments and planning for patients identified to be victims of violence. Collectively these themes highlighted TIC as an essential component of ensuring a safe environment for both patients and staff.

Trauma-informed care was shown to be critical in fostering patients’ physical and emotional safety. Tiller et al detailed safety precautions as a part of a TIC intervention when caring for victims of suspected human trafficking.^40^ These interventions included listing the patient under an alias and discussing with the patient how to prevent the discovery of their location through their mobile device.^40^ However, the most important TIC element of this intervention was empowering the patient to discuss what they thought was best for their safety. This intervention encouraged clinicians to “collaborate with the patient to ensure that we are not jeopardizing safety with our efforts to intervene.”^40^

Staff safety was discussed in three papers,^37^,^40^,^42^ with the most robust intervention being in response to the Boston Marathon bombings in 2014. Lakatos et al was unique in describing TIC and physiological first aid (PFA) interventions for both patients and staff following the Boston Marathon bombings.^42^ Using a TIC and complementary PFA framework they constructed nurse-specific groups and interprofessional groups (including members of chaplaincy, occupational health, nursing leadership, psychiatry, psychology, and social services). These groups were designed to provide support for the variety of ways staff might have been affected by the trauma of the bombings.^42^ The paper emphasizes voluntary supportive services for staff.

Four papers included patient safety assessments,^38^,^40^,^43^,^44^ and two papers specifically focused on incorporation of TIC principles into these assessments.^38^,^43^ Giles et al described how using a TIC framework was foundational in effectively assessing youths at risk for suicide by engaging with them to discuss their hopes, strengths, family support, and ability to practice a safety plan.^43^ As previously discussed, Corbin et al’s work on the Safety, Emotions, Loss, Future model for youths who have experienced violence includes safety as one of the four foundational concepts.^38^

### Additional Themes

Additional interventions emerging from our analysis included the following: conducting trauma screening and assessment^38^,^40^,^43^,^44^; securing leadership buy-in from both hospital and community leaders^35^,^37^,^38^,^4^; developing standardized TIC protocols and programs for vulnerable patient populations^36^,^38^,^40^,^44^; and environmental analysis of the ED.^35^,^37^

## DISCUSSION

Trauma-informed care remains an emerging field in EM with limited operationalization despite positive emergency clinician perceptions of TIC.^45^–^48^ The concepts formally studied that are related to TIC have shown benefit based on initial, but limited, data.^30^–^32^ Our review found 10 studies demonstrating ED interventions explicitly operationalizing a TIC framework. The majority of interventions focused on clinician education and care protocols for historically vulnerable populations (eg, persons impacted by structural racism and oppression). While the data is still preliminary, all included studies showed a positive impact of TIC on either patients or clinicians. Patients reported increased quality of care and increased outpatient referral follow-up rates,^35^ and when experiencing mental health crises spent less time in restraints.^37^ Clinicians reported greater clinical knowledge and comfort when providing care for historically vulnerable patient populations.^35^–^37^,^41^,^47^

Numerous guidelines and best practices for TIC in the ED setting have been published, as we describe in our “Limitations” section. However, operationalization of these best practices and outcomes data remains limited. It may be that the studies are ongoing. For example, the educational interventions included in this review were published between 2014–2020. Most papers included only level 1 and level 2 Kirkpatrick assessments (ie, attitude changes and knowledges gains), and only two included level 3 and level 4 outcomes (ie, clinical practice change and patient outcomes).^49^ The timing of our review may have been insufficient for most groups to collect patient-centered outcomes. Future studies are needed to establish clinician and patient outcomes related to educational TIC interventions in EM.

Our review identified several gaps in the current interventions: lack of universal precautions education; lack of outcomes data; lack of staff-focused interventions; and lack of cost-effectiveness analysis. Across all interventions, both education- and protocol-driven, there was little to no adoption of TIC as a universal precaution for all patients. All interventions captured in our review rely on a population-specific approach (ie, human trafficking, sexual assault, community violence survivors). While this approach may increase clinicians’ awareness of trauma in specific populations, it does not address needs of patients who do not present with “red flags” or who do not present with trauma-related complaints.

Clinicians cannot always predict which patients have experienced adversity; therefore, future educational and programmatic interventions should emphasize TIC as a universal precaution for all-comers.^23^ Education should emphasize that TIC offers the opportunity to avoid trauma related to medical care and interventions itself.^23^ Additionally, only one intervention focused on specifically applying TIC principles to ED staff.^42^ As detailed in the introduction, both clinical and non-clinical ED staff are at high risk for traumatization and re-traumatization based on their work environment.^4^,^5^,^22^ This remains a key area for application of the TIC framework within EM. An increasingly urgent research need is developing in the wake of the COVID-19 pandemic. Future studies with TIC staff-focused interventions would benefit from outcomes data such as validated measures of burnout, PTSD, and STS screening tools.

This review also uncovered a lack of process analysis and environmental analysis of the ED itself. Only two interventions evaluated how the physical space of the ED could be evaluated and improved using a TIC framework.^35^,^37^ None of the interventions examined cost effectiveness or return on investment when TIC models are used, representing another gap in the research. The original studies developing TIC showed no additional cost when the model was employed.^32^ To fully advocate for TIC interventions, especially operational interventions, future studies must include a cost-effectiveness analysis.

The SAMHSA guidelines on TIC include steps for creating trauma-informed institutions and organziations.^12^ Many sectors have adopted these guidelines including social work,^50^ elementary education,^51^ and juvenile justice.^52^ Future studies are needed that analyze the ED from an operational level using a TIC framework. These studies should also include non-clinical ED staff.

## LIMITATIONS

Our paper has several limitations that warrant discussion. Most importantly, by requiring the explicit reference to TIC, we excluded interventions that used principles of TIC without explicitly naming the theory. For example, Cheng et al describe a peer support-based ED violence intervention program in their 2008 paper, and although peer support is one of the six principles of TIC, we did not include the paper in our review as it did not explicitly mention TIC as a guiding framework.^53^ Other violence intervention programs similarly were not included, even though referred to in the literature as “trauma-informed,” because their original publications do not mention TIC.^54^,^55^ Additionally, many papers were excluded due to lack of operationalization of TIC. Many publications described best practices without describing interventions. Guidelines and best practices for TIC care of ED patients experiencing mental health crises,^56^,^57^ sexual assault survivors,^58^–^60^ survivors of community violence,^28^ victims of human trafficking,^61^–^64^ and pediatric patients experiencing trauma^65^ were all excluded due to lack of operationalization. Finally, our search excluded non-English language studies. and we did not conduct a hand search; therefore, we may not have captured all available interventions.

## CONCLUSION

This paper represents the first systematic review of trauma-informed care interventions in the ED setting. The results of the review show that TIC is a small but growing field in the clinical practice of EM. However, an urgent need remains for additional studies to evaluate potential benefits for patients and clinician in the field of EM. With wider adoption of TIC interventions, the ED can be a place of healing for patients and clinicians.

## Supplementary Information







## Figures and Tables

**Table 1:** Data extraction.

Author and date	Country	Specific intervention	Design	Study participants	Number of participants	Focus	Facility type
Educational interventions							

Carter-Snell 2020	Canada	Needs assessment and educational intervention (EESAS)	Participatory action approach	Communities (leaders and stake holders), police, EMS, and ED clinicians	5 Communities, and 290 Clinicians	Sexual assault	Emergency departments, prehospital
Chandramani 2020	United States	Needs assessment and educational intervention (SANE)	Needs assessment, education intervention	EM nurses, residents and attendings	95 (41 Nurses, 34 Residents 20 Attendings)	Sexual assault	An urban academic emergency department
Cole 2014	United States	Educational intervention to decrease use of restraints in ED	Case study	EM nurses and emergency physicians	6 nurses in pilot phase, then “all staff”	Psychologic, mental health	Urban tertiary emergency department
Hoysted 2018	Australia and New Zealand	Web-based training on general TIC principles for pediatric patients	Pilot parallel superiority randomized controlled trial	EM nurses and emergency physicians	71 (65 Nurses, 6	Universal precaution	Emergency departments
Hall 2016	Australia	Modular didactic education on TIC and mental health in ED	Exploratory research with a mixed methods design	EM nurses	34 Nurses	Psychologic, mental health	Emergency department (urban & rural)

TIC-based programs and protocols							

Corbin 2010	United States	Assessment, case management, mentoring, psychoeducational groups, case review	Commentary	Youth (ages 8–30)	NA	Violence	Level 1 trauma center, urban children’s hospital
Giles 2019	United States	TIC assessment and intervention for suicide prevention	Randomized Control trial	Youth	181	Suicide and self harm	Tertiary children’s hospital
Lakatos 2014	United States	TIC response to the Boston Marathon bombings	Commentary	Victims of trauma; clinicians	NA	Violence	Level 1 trauma center
Stolbach 2017	United States	TIC screening, support, education, and intervention	Commentary	Youth	NA	Violence	Pediatric emergency department
Tiller 2020	United States	TIC-based protocol for victims of human trafficking (HEAL Toolkit)	Commentary	High-risk patients for trafficking	NA	Human trafficking	Tertiary emergency department

*EMS*, emergency medical services; *ED*, emergency department; *EM*, emergency medicine; *TIC*, trauma-informed care.

**Table 2:** Major quantitative and qualitative findings for included studies.

Author and date	Specific intervention	Quantitative findings	Qualitative findings
Education			

Carter-Snell 2020	Needs assessment and educational intervention (EESAS)	- Comfort providing sexual assault services significantly improved even at 6-month surveys (P <0.01) in emergency clinicians - Knowledge of consequences of sexual assault, mental health considerations, healthcare interventions, and legal considerations improved post training (P < 0.01)	- Enhanced collaboration across services and issues with ongoing turnover of personnel - Subjective quality of service ratings improved
Chandramani 2020	Needs assessment and educational intervention (SANE)	- Significant improvement in knowledge of elements of assault history 67% to 93% (P < 0.05) and comfort in ability to take history 41% to 86% (P <0.01) in ED residents. -Significant improvement in comfort performing a forensic examination 44% to 87% (P < 0.01) in ED residents.	- In pre-intervention free response, nine participants mentioned a lack of training and education as a barrier to providing better care - Post survey participants expressed that the educational intervention was very helpful to their ability to care for survivors.
Cole 2014	Educational Intervention to decrease use of restraints in ED	- Initially, 15 to 20 episodes of restraints being used per month, which decreased to no episodes by the end of the intervention. - Overall, ED behavioral health seclusion and restraint hours were reduced from 38.5 h/mo to 0 h/mo after 2 years of the program.	-Changing the culture through staff understanding of trauma-informed care was key in improving the patient outcomes. -Success of the program depended upon relationship between ED and behavior health department working together.
Hoysted 2018	Web-based training on general TIC principles for pediatric patients	- Training group had significantly greater knowledge following training and at follow-up than the control (P <.001) - Most participants (74.2%) indicated that the training would be useful in their role in the ED	- Participants liked the online format, found the training to be interesting and informative, and felt the training increased their insight and awareness - Participants stated that there should be more interactive program with the opportunity to practice learned skills
Hall 2016	Modular didactic education on TIC and mental health in ED	- ED nurses reported more confidence in their ability to talk to patients about traumatic experiences (P = 0.001, r = 0.41), respond to disclosures of family violence (P = 0.001, r = 0.41), and understand how their current nursing practice is trauma informed (P = 0.001, r = 0.53)	- Participants had an increased openness to ask questions about trauma and listen to patients’ responses - Participants found the neurobiology component of the education assisted their understanding of trauma
TIC-based programs and protocols			
Corbin 2010	Assessment, case management, mentoring, psychoeducational groups, case review	N/A	Authors concluded a combination of In-hospital peer counseling starting in the ED, outpatient follow-up with home visits to address educational, employment, and behavioral health needs, leads to better all- around care and preventing of future incidents of community violence
Giles 2019	TIC assessment and intervention for suicide prevention	- Patients who received the intervention were significantly more likely to attend outpatient treatment compared with usual care; 79 families (88.8%) received at least one care linkage contact compared to zero in the non-intervention group	- Authors concluded that adding the trauma screening helped to provide trauma-informed care and to link youth directly to trauma- specific, evidenced-based treatments from the ED.
Lakatos 2014	TIC response to the Boston Marathon bombings	N/A	- A team of psychiatric advanced practice nurse using a TIC framework were able to provide comprehensive care to patients, their families, and staff after the Boston Marathon bombings starting in the ED. - Staff reported returning to baseline 3 weeks after the event
Stolbach 2017	TIC screening, support, education, and intervention	N/A	- A TIC-based clinic that first reached out in the ED helped patients recover from the mental harm caused by community violence.
Tiller 2020	TIC-based Protocol for Victims of Human Trafficking (HEAL Toolkit)	N/A	- The development of a TIC standardized protocol ensured that survivors of human trafficking and at-risk patients were treated appropriately and in a standardized manner regardless of the experience of the clinician.

*TIC*, trauma-informed care; *EMS*, emergency medical services; *ED*, emergency department; *EESAS*, Enhanced Emergency Sexual Assault Services; *SANE*, Sexual Assault Nurse Examiners.

**Table 3:** Unique trauma-informed care interventions by theme.

Interventions	Publications including intervention
Education	
Educational needs assessment	35–37, 39
Education through didactic lecture	35–38, 40, 41
Education through online modules	39
Education through standardized patient exercises	36
Tracking clinician outcomes (knowledge, confidence)	35, 36, 37, 39, 41
Tracking patient outcomes	35,37
Education on trauma impacts	35–39, 42
Education on TIC provision for survivors of sexual assault	35, 36
Education on mental health and TIC	37, 41
Education on pediatric traumatic stress	39
Collaboration	
Participatory action model	35
Educational content production collaboration	36, 38
Interprofessional collaboration	35–38, 40, 42
Collaboration between physician specialties	36, 40, 44
Collaboration with community organizations	35, 36,38, 40, 44
Collecting patient perspectives	38
Coordinating outpatient care and follow-up	38, 40, 43, 44
Safety	
Immediate safety assessment	38, 40, 43, 44
Safety planning prior to discharge	38, 40, 43, 44
Trauma screening	38, 40, 43, 44
Psychological first aid for patients and staff	42
Direction to additional resources and appropriate escalation of care	38, 41–43
Enhanced patient privacy	40
ED lockdown with security threat	40
Leadership	
Engage community leaders	35, 38
Engage hospital leadership	37, 42
TIC Protocols	
Violence intervention and prevention programs	38, 44
Human trafficking	40
Environmental Analysis	
Analysis of department layout	35, 37
Analysis of patient care areas	35, 37
Peer support	
Patient peer support groups	38, 42
Staff peer support groups	42

*TIC*, trauma-informed care.
