# Supplementary material for: Trauma-informed Care Interventions in Emergency Medicine: A Systematic Review
Source: West J Emerg Med. 2022 Apr 13;23(3):334–44. doi: 10.5811/westjem.2022.1.53674 (PMC9183774; doi:10.5811/westjem.2022.1.53674)
Supplement: Supplementary file 1 [file wjem-23-334-s001.docx]

Appendix 1: Search Syntax

**PubMed**

(("Emergency Service, Hospital"[Mesh] OR "Emergency Nursing"[Mesh] OR "Social Workers"[Mesh]) OR (Emergency Department*[Title/Abstract] OR Emergency Theatre[Title/Abstract] OR Emergency Center[Title/Abstract] OR Emergency Medical Services[Title/Abstract] OR Emergency Hospital Service*[Title/Abstract] OR Hospital Service Emergency[Title/Abstract] OR Hospital Emergency Service*[Title/Abstract] OR Hospital Emergency Center*[Title/Abstract] OR Emergency Unit*[Title/Abstract] OR Emergency Ward*[Title/Abstract] OR Emergency Outpatient Unit*[Title/Abstract] OR Emergency Room*[Title/Abstract] OR Accident Department[Title/Abstract] OR ER Nurse[Title/Abstract] OR Emergency Room Nursing[Title/Abstract] OR Emergency Room Nurse[Title/Abstract] OR ER Physician[Title/Abstract] OR Emergency Room Physician[Title/Abstract] OR Emergency Room Clinician[Title/Abstract] OR ER Nurse Assistant[Title/Abstract] OR ER Allied Health Professional[Title/Abstract] OR Social Worker*[Title/Abstract] OR Emergency Physician*[Title/Abstract] OR Emergency Nurse Practitioner[Title/Abstract] OR Trauma Nursing[Title/Abstract] OR Case Worker*[Title/Abstract] OR Social Case Worker*[Title/Abstract])) AND (Trauma-informed care[Title/Abstract] OR trauma informed care[Title/Abstract] OR Trauma-informed[Title/Abstract] OR trauma informed[Title/Abstract] OR Trauma[Title/Abstract] AND violence informed care[Title/Abstract] OR Trauma-sensitive[Title/Abstract] OR Trauma awareness[Title/Abstract] OR Trauma-focused[Title/Abstract] OR Trauma centered[Title/Abstract] OR ACE informed[Title/Abstract] OR resilience[Title/Abstract] OR Trauma resilience[Title/Abstract])

**Embase (Elsevier)**

(‘emergency ward’/exp OR ‘emergency physician’/exp OR ‘emergency nurse practitioner’/exp OR ‘emergency nursing’/exp OR ‘social worker’/exp OR ‘emergency department*’:ti,ab,kw OR ‘emergency theatre’:ti,ab,kw OR ‘emergency center’:ti,ab,kw OR ‘emergency medical services’:ti,ab,kw OR ‘emergency hospital service*’:ti,ab,kw OR ‘hospital service emergency’:ti,ab,kw OR ‘hospital emergency service*’:ti,ab,kw OR ‘hospital emergency center*’:ti,ab,kw OR ‘emergency unit*’:ti,ab,kw OR ‘emergency ward*’:ti,ab,kw OR ‘emergency outpatient unit*’:ti,ab,kw OR ‘emergency room*’:ti,ab,kw OR ‘accident department’:ti,ab,kw OR ‘er nurse’:ti,ab,kw OR ‘emergency room nursing’:ti,ab,kw OR ‘emergency room nurse’:ti,ab,kw OR ‘er physician’:ti,ab,kw OR ‘emergency room physician’:ti,ab,kw OR ‘emergency room clinician’:ti,ab,kw OR ‘er nurse assistant’:ti,ab,kw OR ‘er allied health professional’:ti,ab,kw OR ‘social worker*’:ti,ab,kw OR ‘emergency physician*’:ti,ab,kw OR ‘emergency nurse practitioner’:ti,ab,kw OR ‘trauma nursing’:ti,ab,kw OR ‘case worker*’:ti,ab,kw OR ‘social case worker*’:ti,ab,kw) AND (‘trauma informed care’/exp OR ‘resilience’/exp OR ‘trauma-informed care’:ti,ab,kw OR ‘trauma informed care’:ti,ab,kw OR ‘trauma informed’:ti,ab,kw OR (trauma:ti,ab,kw AND ‘violence informed care’:ti,ab,kw) OR ‘trauma sensitive’:ti,ab,kw OR ‘trauma awareness’:ti,ab,kw OR ‘trauma focused’:ti,ab,kw OR ‘trauma centered’:ti,ab,kw OR ‘ace informed’:ti,ab,kw OR resilience:ti,ab,kw OR ‘trauma resilience’:ti,ab,kw)

**Social Services Abstract (ProQuest)**

(MAINSUBJECT.EXACT.EXPLODE("Emergency Medical Services") OR ab(Emergency Department* OR Emergency Theatre OR Emergency Center OR Emergency Medical Services OR Emergency Hospital Service* OR Hospital Service Emergency OR Hospital Emergency Service* OR Hospital Emergency Center* OR Emergency Unit* OR Emergency Ward* OR Emergency Outpatient Unit* OR Emergency Room* OR Accident Department OR ER Nurse OR Emergency Room Nursing OR Emergency Room Nurse OR ER Physician OR Emergency Room Physician OR Emergency Room Clinician OR ER Nurse Assistant OR ER Allied Health Professional OR Social Worker* OR Emergency Physician* OR Emergency Nurse Practitioner OR Trauma Nursing OR Case Worker* OR Social Case Worker*) OR ti(Emergency Department* OR Emergency Theatre OR Emergency Center OR Emergency Medical Services OR Emergency Hospital Service* OR Hospital Service Emergency OR Hospital Emergency Service* OR Hospital Emergency Center* OR Emergency Unit* OR Emergency Ward* OR Emergency Outpatient Unit* OR Emergency Room* OR Accident Department OR ER Nurse OR Emergency Room Nursing OR Emergency Room Nurse OR ER Physician OR Emergency Room Physician OR Emergency Room Clinician OR ER Nurse Assistant OR ER Allied Health Professional OR Social Worker* OR Emergency Physician* OR Emergency Nurse Practitioner OR Trauma Nursing OR Case Worker* OR Social Case Worker*)) AND (ti(Trauma-informed care OR trauma informed care OR Trauma-informed OR trauma informed OR Trauma AND violence informed care OR Trauma-sensitive OR Trauma awareness OR Trauma-focused OR Trauma centered OR ACE informed OR resilience OR Trauma resilience) OR ab(Trauma-informed care OR trauma informed care OR Trauma-informed OR trauma informed OR Trauma AND violence informed care OR Trauma-sensitive OR Trauma awareness OR Trauma-focused OR Trauma centered OR ACE informed OR resilience OR Trauma resilience))

**PsycInfo (EBSCO)**

(TI ( Emergency Department* OR Emergency Theatre OR Emergency Center OR Emergency Medical Services OR Emergency Hospital Service* OR Hospital Service Emergency OR Hospital Emergency Service* OR Hospital Emergency Center* OR Emergency Unit* OR Emergency Ward* OR Emergency Outpatient Unit* OR Emergency Room* OR Accident Department OR ER Nurse OR Emergency Room Nursing OR Emergency Room Nurse OR ER Physician OR Emergency Room Physician OR Emergency Room Clinician OR ER Nurse Assistant OR ER Allied Health Professional OR Social Worker* OR Emergency Physician* OR Emergency Nurse Practitioner OR Trauma Nursing OR Case Worker* OR Social Case Worker* ) OR AB ( Emergency Department* OR Emergency Theatre OR Emergency Center OR Emergency Medical Services OR Emergency Hospital Service* OR Hospital Service Emergency OR Hospital Emergency Service* OR Hospital Emergency Center* OR Emergency Unit* OR Emergency Ward* OR Emergency Outpatient Unit* OR Emergency Room* OR Accident Department OR ER Nurse OR Emergency Room Nursing OR Emergency Room Nurse OR ER Physician OR Emergency Room Physician OR Emergency Room Clinician OR ER Nurse Assistant OR ER Allied Health Professional OR Social Worker* OR Emergency Physician* OR Emergency Nurse Practitioner OR Trauma Nursing OR Case Worker* OR Social Case Worker* ) OR KW ( Emergency Department* OR Emergency Theatre OR Emergency Center OR Emergency Medical Services OR Emergency Hospital Service* OR Hospital Service Emergency OR Hospital Emergency Service* OR Hospital Emergency Center* OR Emergency Unit* OR Emergency Ward* OR Emergency Outpatient Unit* OR Emergency Room* OR Accident Department OR ER Nurse OR Emergency Room Nursing OR Emergency Room Nurse OR ER Physician OR Emergency Room Physician OR Emergency Room Clinician OR ER Nurse Assistant OR ER Allied Health Professional OR Social Worker* OR Emergency Physician* OR Emergency Nurse Practitioner OR Trauma Nursing OR Case Worker* OR Social Case Worker* ) OR (DE “Emergency Medicine”) OR MA ( “Emergency Service, Hospital”[Mesh] OR “Emergency Nursing”[Mesh] OR “Social Workers”[Mesh] )) AND (( ((DE “Trauma-Informed Care”) OR (DE “Trauma Treatment”)) OR (DE “Resilience (Psychological)”) ) OR TI ( Trauma-informed care OR trauma informed care OR Trauma-informed OR trauma informed OR Trauma and violence informed care OR Trauma-sensitive OR Trauma awareness OR Trauma-focused OR Trauma centered OR ACE informed OR resilience OR Trauma resilience ) OR AB ( Trauma-informed care OR trauma informed care OR Trauma-informed OR trauma informed OR Trauma and violence informed care OR Trauma-sensitive OR Trauma awareness OR Trauma-focused OR Trauma centered OR ACE informed OR resilience OR Trauma resilience ) OR KW ( Trauma-informed care OR trauma informed care OR Trauma-informed OR trauma informed OR Trauma and violence informed care OR Trauma-sensitive OR Trauma awareness OR Trauma-focused OR Trauma centered OR ACE informed OR resilience OR Trauma resilience ))

**CINAHL (EBSCO)**

((TI ( Emergency Department* OR Emergency Theatre OR Emergency Center OR Emergency Medical Services OR Emergency Hospital Service* OR Hospital Service Emergency OR Hospital Emergency Service* OR Hospital Emergency Center* OR Emergency Unit* OR Emergency Ward* OR Emergency Outpatient Unit* OR Emergency Room* OR Accident Department OR ER Nurse OR Emergency Room Nursing OR Emergency Room Nurse OR ER Physician OR Emergency Room Physician OR Emergency Room Clinician OR ER Nurse Assistant OR ER Allied Health Professional OR Social Worker* OR Emergency Physician* OR Emergency Nurse Practitioner OR Trauma Nursing OR Case Worker* OR Social Case Worker* ) OR AB ( Emergency Department* OR Emergency Theatre OR Emergency Center OR Emergency Medical Services OR Emergency Hospital Service* OR Hospital Service Emergency OR Hospital Emergency Service* OR Hospital Emergency Center* OR Emergency Unit* OR Emergency Ward* OR Emergency Outpatient Unit* OR Emergency Room* OR Accident Department OR ER Nurse OR Emergency Room Nursing OR Emergency Room Nurse OR ER Physician OR Emergency Room Physician OR Emergency Room Clinician OR ER Nurse Assistant OR ER Allied Health Professional OR Social Worker* OR Emergency Physician* OR Emergency Nurse Practitioner OR Trauma Nursing OR Case Worker* OR Social Case Worker* ) OR ( ((MH "Social Work") OR (MH "Emergency Nurse Practitioners") OR (MH "Physicians, Emergency") OR (MH "Emergency Service+") OR (MH "Emergency Nursing+"))) AND (TI ( Trauma-informed care OR trauma informed care OR Trauma-informed OR trauma informed OR Trauma and violence informed care OR Trauma-sensitive OR Trauma awareness OR Trauma-focused OR Trauma centered OR ACE informed OR resilience OR Trauma resilience ) OR AB ( Trauma-informed care OR trauma informed care OR Trauma-informed OR trauma informed OR Trauma and violence informed care OR Trauma-sensitive OR Trauma awareness OR Trauma-focused OR Trauma centered OR ACE informed OR resilience OR Trauma resilience )))
