# Supplementary material for: Trauma-informed Care Interventions in Emergency Medicine: A Systematic Review
Source: West J Emerg Med. 2022 Apr 13;23(3):334–44. doi: 10.5811/westjem.2022.1.53674 (PMC9183774; doi:10.5811/westjem.2022.1.53674)
Supplement: Supplementary file 2 [file wjem-23-334-s002.docx]

**Appendix 2: Trauma-Informed Care**

The Substance Abuse and Mental Health Services Administration defines trauma as “an event, series of events, or set of circumstances that is experienced by an individual as physically or emotionally harmful or threatening and that has lasting adverse effects.”^1^ This includes sexual, physical, and verbal abuse, neglect, racism, oppression, homophobia, sexism, and bullying.

Trauma is linked with poor health, in children and in adults. The Adverse Childhood Experiences (ACE) study found high rates of trauma among a predominantly white, middle-class, and insured study population.^2^ The study showed a strong dose-response relationship between childhood trauma and adult heart, lung, and liver disease, obesity, diabetes, depression, substance abuse, sexually transmitted infection risk, and intimate partner violence.^2^

In response to the growing body of evidence surrounding trauma and health impacts, the Substance Abuse and Mental Health Services Administration (SAMHSA) developed the framework of trauma-informed care (TIC) to guide clinicians and institutions in providing care that resists retraumatization and promotes strength and healing.^1^

According to SAMHSA’s concept of a trauma-informed approach, “A program, organization, or system, that is trauma informed:

- Realizes the widespread impact of trauma and understands potential paths for recovery;
- Recognizes the signs and symptoms of trauma in clients, families, staff and others involved in the system;
- Responds by fully integrating knowledge about trauma into policies, procedures, and practices;
- Seeks to actively resist re-traumatization.”^1^

This approach centers around six principles: : 1) safety; 2) trustworthiness and transparency; 3) peer support; 4) collaboration and mutuality; 5) empowerment, voice, and choice; 6) and cultural, historical, and gender issues. Application of these principles to clinical care has been adopted in nursing, behavioral health, primary care, and dentistry.^3^

Practically, the principles of primary care can be applied to all essential elements of clinical care including history taking, physical exam, and procedures.^4–6^ During history taking clinicians can attend to safety by introducing themselves and asking for a patient’s name and pronouns. Clinicians can attend to collaboration by co-developing the follow-up care plan with patients. An emphasis on empowerment leaves space for patients to disagree with treatment plans and opt out of answering questions at any time.

When applied to the physical exam, TIC utilizes the same 6 principles described above. To ensure safety, the clinician should not to physically block the door of the exam room. Clinicians should ensure the patient is always aware of their presence in space and consider using an anterior approach to thyroid examination. Emphasizing collaboration may include inquiring about previous experiences with an examination and adjusting based on patient preference.

1. *SAMHSA’s Concept of Trauma and Guidance for a Trauma-Informed Approach*. Substance Abuse & Mental Health Services Administration; 2014:27.

2. Felitti VJ, Anda RF, Nordenberg D, et al. Relationship of childhood abuse and household dysfunction to many of the leading causes of death in adults. The Adverse Childhood Experiences (ACE) Study. *Am J Prev Med*. 1998;14(4):245-258. doi:10.1016/s0749-3797(98)00017-8

3. *Trauma-Informed Care in Behavioral Health Services. Treatment Improvement Protocol (TIP) Series 57.* Substance Abuse and Mental Health Services Administration; 2014.

4. Elisseou S, Puranam S, Nandi M. A Novel, Trauma-Informed Physical Examination Curriculum for First-Year Medical Students. *MedEdPORTAL*. 2019;15:10799. doi:10.15766/mep_2374-8265.10799

5. Brown T, Mehta PK, Berman S, et al. A Trauma-Informed Approach to the Medical History: Teaching Trauma-Informed Communication Skills to First-Year Medical and Dental Students. *MedEdPORTAL*. 2021;17(1):11160. doi:10.15766/mep_2374-8265.11160

6. Raja S, Hoersch M, Rajagopalan CF, Chang P. Treating patients with traumatic life experiences. *The Journal of the American Dental Association*. 2014;145(3):238-245. doi:10.14219/jada.2013.30
