# Supplementary material for: Trauma-informed Care Interventions in Emergency Medicine: A Systematic Review
Source: West J Emerg Med. 2022 Apr 13;23(3):334–44. doi: 10.5811/westjem.2022.1.53674 (PMC9183774; doi:10.5811/westjem.2022.1.53674)
Supplement: Supplementary file 3 [file 53674_Appendix_3.docx]

# **Appendix 3. Newcastle-Ottawa Quality Assessment Scale and Critical Appraisal Skills Tool**

*We chose the Newcastle-Ottawa Quality Assessment Scale and Critical Appraisal Skills Programme (CASP) checklist to assess the quality of each included study. The Newcastle-Ottawa tool was adopted because it provided the capability to appraise cohort, cross-sectional, and case-control studies. The CASP Checklist was used to assess the quality of all qualitative studies. No papers were excluded based upon this assessment.*

**Scoring System**

For the Newcastle-Ottawa Criteria the associated start system was used to assess the quality of the study. If a paper met the minimum of 3 stars total it was included, with a minimum of one per section. For the CASP checklist a scoring system to assess if the paper was on low quality and to therefore exclude. Points were awarded to each mark of quality: Yes = 2 No = -2 Unclear =-1 N/A = 0 If a paper identified a confounding factor which was not dealt with (stratification) it was completely excluded.

Papers score > or including 0 include

Papers score < 0 exclude

score < 0 = very low; 0 ≤ score < 5 = low; 5 ≤ score < 10 = moderate; score ≥ 10 = strong

| **Study** | **Selection** | | | | | **Comparability** | **Exposure** | | |  | **Total** |
| --- | --- | --- | --- | --- | --- | --- | --- | --- | --- | --- | --- |
|  | *Is the case definition adequate?* | *Representativeness of the cases* | *Selection of Controls* | *Definition of Controls* | *Total* | *Comparability of cases and controls on the basis of the design or analysis* | *Ascertainment of exposure* | *Same method of ascertainment for cases and controls* | *Non-Response rate* | *Total* |  |
| Hoysted 2018 | A* | A* | A* | A* | 4 | Yes* | A* | A* | B | 3 | 7 |
| Giles 2019 | B | B | B | A* | 1 | Yes* | C | A* | B | 2 | 3 |

Newcastle-Ottawa RCT Checklist

Newcastle-Ottawa Criteria for Cohort Studies

| **Study** | **Selection** | | | | | **Comparability** | **Outcome** | | |  | **Total** |
| --- | --- | --- | --- | --- | --- | --- | --- | --- | --- | --- | --- |
|  | *Representativeness of the exposed cohort* | *Selection of the non exposed cohort* | *Ascertainment of exposure* | *Demonstration that outcome of interest was not present at start of study* | *Total* | *Comparability of cohorts on the basis of the design or analysis* | *Assessment of outcome* | *Was follow-up long enough for outcomes to occur* | *Adequacy of follow up of cohorts* | *Total* |  |
| Cole 2014 | A* | C | A* | A* | 3 | Yes* | B* | A* | B | 3 | 6 |

CASP Critical Appraisal Checklist

|  | *Was there a clear  statement of the aims of  the research?* | *Is a qualitative  methodology  appropriate?* | *Was the research  design appropriate to  address the aims of the  research?* | *Was the recruitment  strategy appropriate to  the aims of the  research?* | *Was the data collected in  a way that addressed the  research issue?* | *Has the relationship  between researcher and  participants been  adequately considered?* | *Have ethical issues been  taken into consideration?* | *Was the data analysis  sufficiently rigorous?* | *Is there a clear statement  of findings?* | *How valuable is the  research?* | *Total* |
| --- | --- | --- | --- | --- | --- | --- | --- | --- | --- | --- | --- |
| Corbin 2010 | 2 | 2 | 2 | 2 | -2 | -1 | 2 | 2 | 2 | 2 | 13 |
| Lakatos 2014 | 2 | -2 | -1 | 2 | 2 | 2 | 2 | -1 | 2 | 2 | 10 |
| Stolbach 2017 | 2 | -2 | 2 | -1 | 2 | 2 | 2 | -2 | 2 | -1 | 8 |
| Tiller 2020 | 2 | -2 | 2 | 2 | 2 | 2 | 2 | 2 | 2 | 2 | 16 |
| Carter-Snell 2020 | 2 | -1 | 2 | 2 | 2 | 2 | 2 | 2 | 2 | 2 | 19 |
| Hall 2016 | 2 | -2 | 2 | -1 | 2 | 2 | 2 | -2 | 2 | 2 | 15 |
